# Reference values for fetal tissue velocity imaging and a new approach to evaluate fetal myocardial function

**DOI:** 10.1186/1476-7120-11-29

**Published:** 2013-08-16

**Authors:** Nina NE Elmstedt, Jonas JJ Johnson, Britta BL Lind, Kjerstin KFW Ferm-Widlund, Lotta LH Herling, Magnus MW Westgren, Lars-Åke LAB Brodin

**Affiliations:** 1 Department of Medical Engineering, School of Technology and Health, KTH Royal Institute of Technology, Alfred Nobels Allé 10, Huddinge 141 52, Sweden; 2 Department of Obstetrics and Gynecology, Centre of Fetal Medicine, Karolinska University Hospital, Kirurggatan 9, Huddinge 141 86, Sweden

**Keywords:** Color-coded tissue velocity imaging, Fetus, Myocardium, Normal reference values, Cardiac state diagram, Atrioventricular plane displacement, Myocardial time interval, Longitudinal myocardial peak velocity

## Abstract

**Objectives:**

Myocardial function can be evaluated using color-coded tissue velocity imaging (TVI) to analyze the longitudinal myocardial velocity profile, and by expressing the motion of the atrioventricular plane during a cardiac cycle as coordinated events in the cardiac state diagram (CSD). The objective of this study was to establish gestational age specific reference values for fetal TVI measurements and to introduce the CSD as a potential aid in fetal myocardial evaluation.

**Methods:**

TVI recordings from 125 healthy fetuses, at 18 to 42 weeks of gestation, were performed with the transducer perpendicular to the apex to provide a four-chamber view. The myocardial velocity data was extracted from the basal segment of septum as well as the left and right ventricular free wall for subsequent offline analysis.

**Results:**

During a cardiac cycle the longitudinal peak velocities of septum increased with gestational age, as did the peak velocities of the left and right ventricular free wall, except for the peak velocity of post ejection. The duration of rapid filling and atrial contraction increased during pregnancy while the duration of post ejection decreased. The duration of pre ejection and ventricular ejection did not change significantly with gestational age.

**Conclusion:**

Evaluating fetal systolic and diastolic performance using TVI together with CSD could contribute to increase the knowledge and understanding of fetal myocardial function and dysfunction. The pre and post ejection phases are the variables most likely to indicate fetuses with abnormal myocardial function.

## Introduction

In the progression of fetal hemodynamic adaptation, the assessment of fetal myocardial function could be of crucial importance when evaluating fetuses at risk or suffering from intrauterine hypoxia [1,2]. Although this has been common knowledge since many years, myocardial performance is typically evaluated by specialists in fetal cardiology, and methods to perform these evaluations are regarded as rather cumbersome and have consequently not been introduced in clinical practice. Furthermore, most studies on myocardial performance have until now been based on conventional Doppler, which measures global cardiac function restricted to flow measurements [3-7]. Color-coded tissue velocity imaging (TVI) offers quantitative measurements of regional myocardial function, and can potentially provide more sensitive and thus earlier indications of myocardial dysfunction. TVI enables adequate temporal resolution (>200 frames/s) [8] unlike speckle tracking (<80 frames/s) [9] and doesn’t overestimate true motion or alter offline gain settings in contrast to spectral Doppler [10]. Velocity can also be measured simultaneously in the different myocardial walls during the same cardiac cycle.

**Figure 1:**
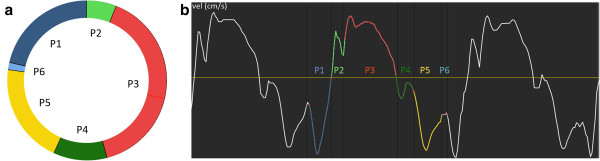
**The cardiac state diagram (CSD).** Example displaying **(a)** a cardiac state diagram CSD of septum from a healthy fetus at 36 weeks of gestation, and **(b)** the extracted myocardial velocity (thick curve) and acceleration (thin curve) from which the CSD was generated.

The cardiac state diagram (CSD) is a new visualization tool for the pumping and regulating function of the heart [11] that provides quantitative analysis of the timing of mechanical events in the cardiac cycle without the need of a concurrent ECG signal. Based on the hypothesis that the heart’s pumping and regulating function acts according to the Dynamic Adaptive Piston Pump (DAPP) [12], this tool presents comparative data of systolic and diastolic performance and improve separate assessment thereof - providing a more complete overview of the cardiac function [13]. The different time intervals during a cardiac cycle are displayed in a circular diagram, a visual interpretation of which can be seen in Figure 1. To be able to understand fetal cardiac physiology it is essential to understand fetal myocardial function and development throughout gestation. Thus, the general objective of this study was to establish normal reference ranges and gestational age related changes of fetal TVI measurements and to introduce the CSD as a potential aid in fetal myocardial evaluation.

## Methods

The study included 125 fetuses, at a gestational age of 18 to 42 weeks, all referred to the Centre of Fetal Medicine at the Karolinska University Hospital Huddinge during 2009–2012. Gestational age was determined according to ultrasound in the 16-18th week of gestation. Characteristics of the study population are presented in Table 1. All women enrolled were healthy, and experienced a normal singleton pregnancy. Twin pregnancies and in vitro fertilization (IVF) were excluded. The study was approved by the Regional Ethics committee of Stockholm, Sweden, and all subjects gave their informed consent to participate.

**Table 1:** Characteristics of the studied fetal population

**Number of subjects**	**Sex female/male**	**GA**	**GA at delivery**	**MA**	**BW**
125	62/63	30 (18–42)	40 (38–42)	30 (19–46)	3525 (2538–4815)

*GA,* Gestational age (weeks); *MA,* Maternal age (years) and *BW,* Birth weight (g) presented as median (range).

**Figure 2:**
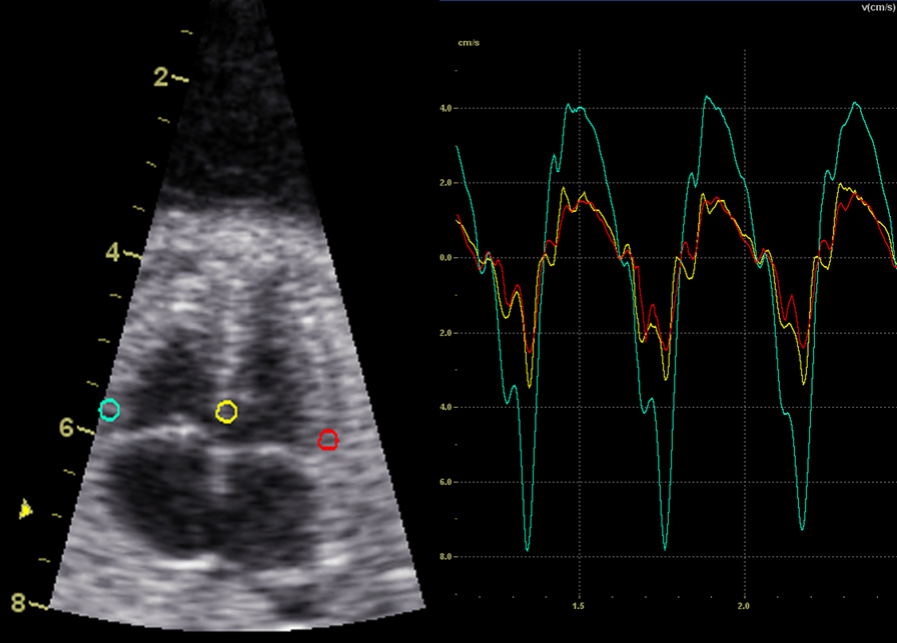
**Tissue velocity imaging.** Example displaying the longitudinal velocity profile extracted from the basal segment of septum (yellow) as well as the left (red) and right ventricular free wall (turquoise).

Tissue Doppler echocardiography data was obtained with a GE Vivid-i equipment (GE Vingmed, Norway), using a 3S-RS transducer. All recordings were performed with the transducer perpendicular to the apex, providing a view of the fetal myocardium equivalent to an apical four-chamber view*.* The 2D and TVI sector widths were minimized to obtain a frame rate > 200 frames/s (median 209 frames/s, range 200 to 273), which is necessary for adequate reconstruction of TVI data for the fetal myocardium [8]. The recordings were performed by an experienced midwife and stored as cine loops of five to 10 consecutive cardiac cycles for offline analysis using EchoPAC (GE Vingmed, Norway) and GHLab (Gripping Heart AB, Sweden). Longitudinal myocardial tissue velocity was extracted from fixed region of interest (ROI) in the basal segment of septum as well as the left and right ventricular free wall during the end of systole in the same cardiac cycle, apical to the atrioventricular plane (AV-piston), as illustrated in Figure 2. The ROI was set between 1–3 mm, depending on image characteristics such as thickness of the myocardial wall and interference from valve motion or fetal/maternal movement. According to DAPP technology the term AV-piston was used instead of the conventional term AV-plane, better describing the cardiac mechanics.

The cardiac cycle was divided into six main phases: atrial contraction, pre ejection, ventricular ejection, post ejection, rapid filling and slow filling. Systole was defined as pre ejection and ventricular ejection, while diastole was defined as post ejection, rapid filling, atrial contraction and slow filling. The time intervals were measured according to the changes from static to dynamic work, based on the motion shifts of the AV-piston, in compliance with DAPP technology. This makes identification of the time intervals possible without a concurrent ECG signal. The measuring points are slightly different from prevalent measuring points, as the phases of pre and post ejection are defined according to heart mechanics unlike the isovolumetric contraction and relaxation, identified based on the zero axis crossing points of the velocity curve. This is usually carried out in combination with reference points in the ECG signal, and the occasional anatomical M-mode image, in order to acquire a precise electrophysiological timing. This is not possible when screening the fetus, which precludes accurate timing of the mechanical events. The phases are displayed in Figure 1 and the time intervals defined as follows.

Atrial contraction begins with an elevation of the AV-piston, during which tension forces are created in the ventricular walls. When these tension forces become greater than the contraction forces in the atria the AV-piston start to move down towards the apex and the atrial contraction phase ends. This ventricular reshaping together with the vortex formations created in the flow behind the atrioventricular valves initiate the closure of the atrioventricular valves and is the beginning of pre ejection - the transition phase (volume to tension) between atrial contraction and ventricular ejection. During pre ejection the atrioventricular valves close and the interventricular pressure rises. This phase ends with a powerful tension increase as the acceleration of blood starts to initiate the opening of the semilunar valves and ventricular ejection begins. The interventricular pressure exceeds the pressure in the aorta and the pulmonary artery, causing the semilunar valves to open and blood to be ejected into the systematic circulation. Ventricular ejection ends when there is no outflow from the semilunar valves and they are about to close just before a backflow into the ventricles is generated. This initiates post ejection – the transition phase (tension to volume) between ventricular ejection and rapid filling. During post ejection the ventricular myocardium starts to relax. The forces of the backflow overcome the tension in the muscles and the ventricular volumes increase. Rapid filling starts as the atrioventricular valves are about to open and a reverse volumetric reshaping process begins. At that time the interventricular pressure drops, muscle tension decrease and the AV-piston returns to its neutral position by the inflow controlled hydraulic return. This phase ends as soon as the dynamic and resilient forces outside the heart have ceased and slow filling begins. Static and dynamic forces generated by blood flow into the heart are now the only forces left to keep the heart in an expanded position. Once the slow filling phase ends a new cardiac cycle begins.

The data set was analyzed using SPSS (PASW Statistics 18). Linear regression analyses were displayed in scatter plots as well as in tables presented as mean values with 95% confidence interval. For the variables of insignificant change, the results were presented as median values within a range.

## Results

The myocardial time intervals and velocities measured during a cardiac cycle, as well as the Tei index and the e’/a’ ratio, extracted from septum as well as the left and right ventricular free wall, are presented in Table 2. The variables that were observed to significantly change throughout pregnancy are further described with gestational age specific mean values and confidence intervals in Table 3.

**Table 2:** The mean values from 18 weeks of gestation to term for the myocardial time intervals and velocities during a cardiac cycle, as well as the Tei index and e’/a’ ratio

**Variable**	**Abbreviation**	**Septum**	**Left ventricular free wall**	**Right ventricular fee wall**
**Time intervals (ms)**				
Atrial contraction	P1	67 / 83 (r = 0,344)	61 / 84 (r = 0,317)	81 / 94 (r = 0,214)
Pre ejection	P2	23 (11–56)	24 (9–54)	26 (0–76)
Ventricular ejection	P3	180 (95–277)	184 (126–245)	182 (95–303)
Post ejection	P4	63 / 41 (r = 0,441)	61 / 36 (r = 0,353)	49 (17–87)
Rapid filling	P5	71 / 109 (r = 0,403)	74 / 100 (r = 0,393)	72 / 98 (r = 0,416)
Slow filling	P6	8 / 110 (r = 0,287 )	4 (0–44)	3 (0–36)
Tei index		0,42 (0,13 – 0,64)	0,41 (0,17 – 0,85)	0,41 (0,14 – 0,89)
**Peak velocities (cm/s)**				
Atrial contraction	a’	−2,4 / –3,9 (r = 0,387)	−1,9 / –4,0 (r = 0,375)	−4,0 / −6,2 (r = 0,421)
Pre ejection (pos)	pre’_pos_	0,4 / 1,1 (r = 0,427)	0,3 / 2,0 (r = 0,489)	0,7 / 3,0 (r = 0,553)
Pre ejection (neg)	pre’_neg_	−0,1 (−2,8 – 1,5)	−0,1 (−1,3 – 0,3)	−0,1 (−1,3 – 3,7)
Ventricular ejection	s’	1,5 / 2,9 (r = 0,566)	1,1/ 2,8 (r = 0,512)	1,7 / 4,7 (r = 0,599)
Post ejection (neg)	post’_neg_	−0,3 (−1,8 – 1,0)	−0,3 (−4,2 – o,9)	−0,4 (−6,9 – 0,8)
Post ejection (pos)	post’_pos_	0,3 / 1,0 (r = 0,395)	0,3 (−0,8 – 2,8)	0,4 (−5,0 – 2,7)
Rapid filling	e’	−1,3 / −3,3 (r = 0,509)	−1,2 / −3,1 (r = 0,528)	−1,4 / −4,6 (r = 0,527)
Slow filling	p6’	−0,5 / −1,3 (r = 0,373)	−0,7 (−4,9 – 2,8)	−1,9 (−6,7 – 3,5)
e’/a’		0,54 / 0,84 (r = 0,316)	0,56 / 0,84 (r = 0,358)	0,45 / 0,79 (r = 0,438)

The variables that significantly changed throughout gestation are presented as mean values, where the nominator represents the mean value at 18 weeks of gestation and the denominator represents the mean value at term. The r-value is Pearson’s correlation coefficient, the correlation to advancing gestational age. The variables that did not show any significant change with gestational age are presented as median values within a range.

**Table 3:** Gestational age specific reference values for fetal myocardial TVI measurements of longitudinal velocities and time intervals during a cardiac cycle

**GA (weeks)**	**18–21**	**22–25**	**26–29**	**30–33**	**34–37**	**38–42**
Subjects	20	20	20	20	20	25
	**Time intervals (ms) mean (95% CI)**					
***Septum***						
P1	66 (62 – 70)	72 (68 – 76)	80 (70 – 89)	80 (72 – 89)	82 (74 – 90)	83 (78 – 89)
P4	58 (53 – 64)	54 (50 – 59)	55 (48 – 61)	53 (47 – 59)	49 (44 – 55)	41 (34 – 49)
P5	69 (57 – 82)	74 (68 – 79)	81 (69 – 93)	80 (67 – 93)	82 (71 – 92)	110 (96 – 123)
P6	8 (5–11)	5 (3–7)	5 (3–7)	4 (3–6)	4 (2–6)	3 (1 – 5)
***Left ventricular free wall***						
P1	66 (62 – 70)	69 (66 – 71)	75 (68 – 82)	74 (67 – 82)	78 (72 – 84)	81 (77 – 86)
P4	62 (57 – 67)	55 (50 – 61)	57 (51 – 64)	45 (40 – 51)	43 (36 – 51)	36 (30 – 41)
P5	67 (56 – 77)	74 (67 – 81)	80 (71 – 89)	81 (73 – 89)	85 (75 – 95)	100 (86 – 114)
***Right ventricular free wall***						
P1	78 (73 – 83)	87 (82 – 93)	88 (78 – 98)	94 (83 – 103)	94 (82 – 106)	96 (85 – 108)
P5	61 (51 – 71)	70 (63 – 76)	72 (60 – 85)	69 (60 – 77)	92 (77 – 107)	99 (83 – 114)
	**Peak velocities (cm/s) mean (95% CI)**					
***Septum***						
a’	–2,5 (1,3 – 2,8)	–2,9 (2,5 – 3,2)	–3,2 (2,7 – 3,7)	–3,7 (3,3 – 4,0)	–3,4 (2,8 – 3,9)	–3,8 (3,3 – 4,3)
pre’_pos_	0,4 (0,3 – 0,6)	0,4 (0,2 – 0,6)	0,3 (0,2 – 0,5)	0,7 (0,4 – 0,9)	0,7 (0,4 – 0,9)	1,1 (0,8 – 1,4)
s’	1,6 (1,3 – 1,8)	2,0 (1,8 – 2,3)	2,0 (1,8 – 2,2)	2,3 (2,2 – 2,4)	2,6 (2,2 – 3,0)	2,9 (2,6 – 3,3)
post’_pos_	0,2 (0,1 – 0,3)	0,2 (0,0 – 0,3)	0,4 (0,2 – 0,5)	0,4 (0,2 – 0,7)	0,2 (0,0 – 0,4)	1,0 (0,7 – 1,3)
e’	–1,4 (1,2 – 1,6)	–2,2 (1,9 – 2,4)	–2,5 (2,1 – 2,8)	–2,7 (2,5 – 3,0)	–2,8 (2,3 – 3,2)	–3,2 (2,6 – 3,8)
p6’	–0,5 (0,8 – 0,2)	–0,7(1,0 – 0,4)	–0,8 (1,1 – 0,5)	–1,6 (1,9 – 1,2)	–1,2 (1,6 – 0,8)	–1,3 (1,7 – 0,9)
***Lefts ventricular free wall***						
a’	–2,0 (1,7 – 2,2)	–3,1 (2,8 – 3,5)	–3,9 (3,2–4,6)	–4,4 (3,3 – 5,0)	–3,4 (2,9 – 3,8)	–3,9 (3,2 – 4,6)
pre’_pos_	0,3 (0,2 – 0,4)	0,5 (0,3 – 0,7)	0,6 (0,3 – 0,8)	0,8 (0,4 – 1,2)	1,2 (0,8 – 1,6)	1,9 (1,1 – 2,7)
s’	1,3 (1,1 – 1,5)	1,9 (1,7 – 2,2)	2,3 (2,0 – 2,7)	2,4 (2,2 – 2,6)	2,9 (2,4 – 3,4)	2,9 (2,4 – 3,3)
e’	–1,3 (1,1 – 1,6)	–2,1 (1,7 – 2,4)	–2,6 (2,1 – 3,1)	–3,6 (3,1 – 4,1)	–3,2 (2,8 – 3,6)	–3,0 (2,6 – 3,5)
***Right ventricular free wall***						
a’	–4,4 (3,8 – 5,0)	–5,2 (4,8 – 5,7)	–5,6 (4,9 – 6,4)	–6,5 (5,9 – 7,1)	–6,7 (5,8 – 7,7)	–6,0 (5,3 – 6,7)
pre’_pos_	0,6 (0,4 – 0,9)	1,0 (0,7 – 1,4)	1,3 (0,8 – 1,8)	1,3 (0,8 – 1,9)	2,2 (1,5 – 2,9)	3,0 (2,2 – 3,8)
s’	1,8 (1,5 – 2,2)	2,7 (2,3 – 3,1)	3,1(2,6 – 3,7)	3,4 (3,1 – 3,7)	4,2(3,6 – 4,7)	4,5 (3,6 – 5,4)
e’	–1,7 (1,1 – 2,2)	–3,3 (2,8 – 3,7)	–4.2 (3,5 – 4,9)	–4,9 (4,3 – 5,5)	–5,6 (4,6 – 6,5)	–4,5 (3,6 – 5,3)

The results from the linear regression analyses are presented as mean values with 95% confidence intervals. **P1** and **a’**, atrial contraction; **pre’**_**pos**_**,** pre ejection; **s’,** ventricular ejection; **P4 and post’**_**pos**_**,** post ejection; **P5** and **e’,** rapid filling; **P6** and **p6’,** slow filling.

**Figure 3:**
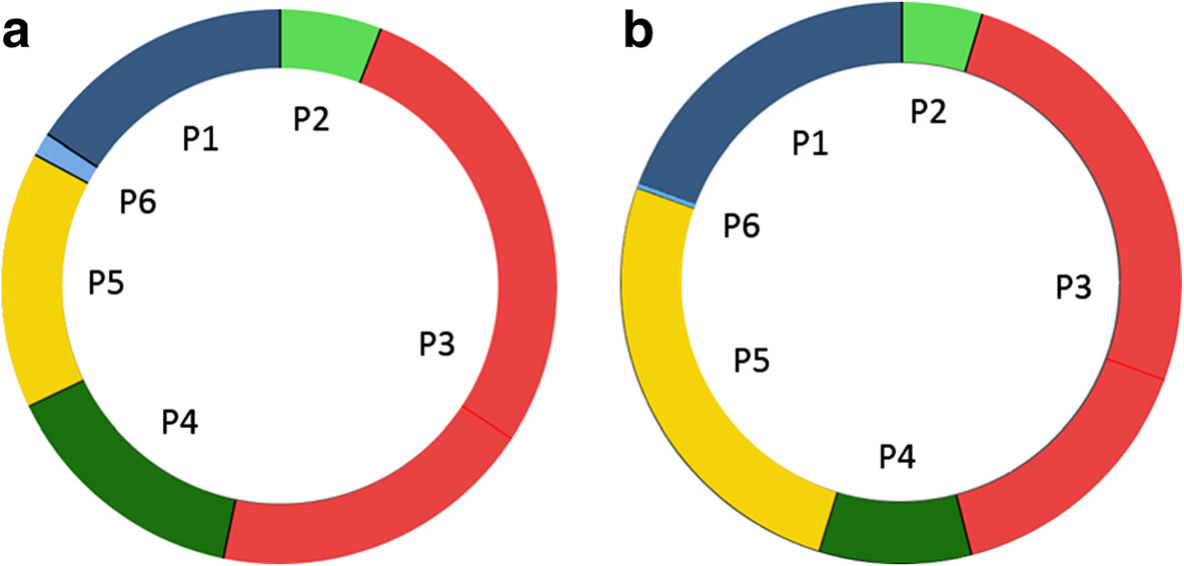
**The cardiac state diagram (CSD).** Example displaying the cardiac state diagram (CSD) generated from septum, representing the average fetus **(a)** at 18 weeks of gestation and **(b)** at term. P1, atrial contraction; P2, pre ejection; P3, ventricular ejection; P4, post ejection; P5, rapid filling; P6, slow filling. Full circle represents one cardiac cycle.

**Figure 4:**
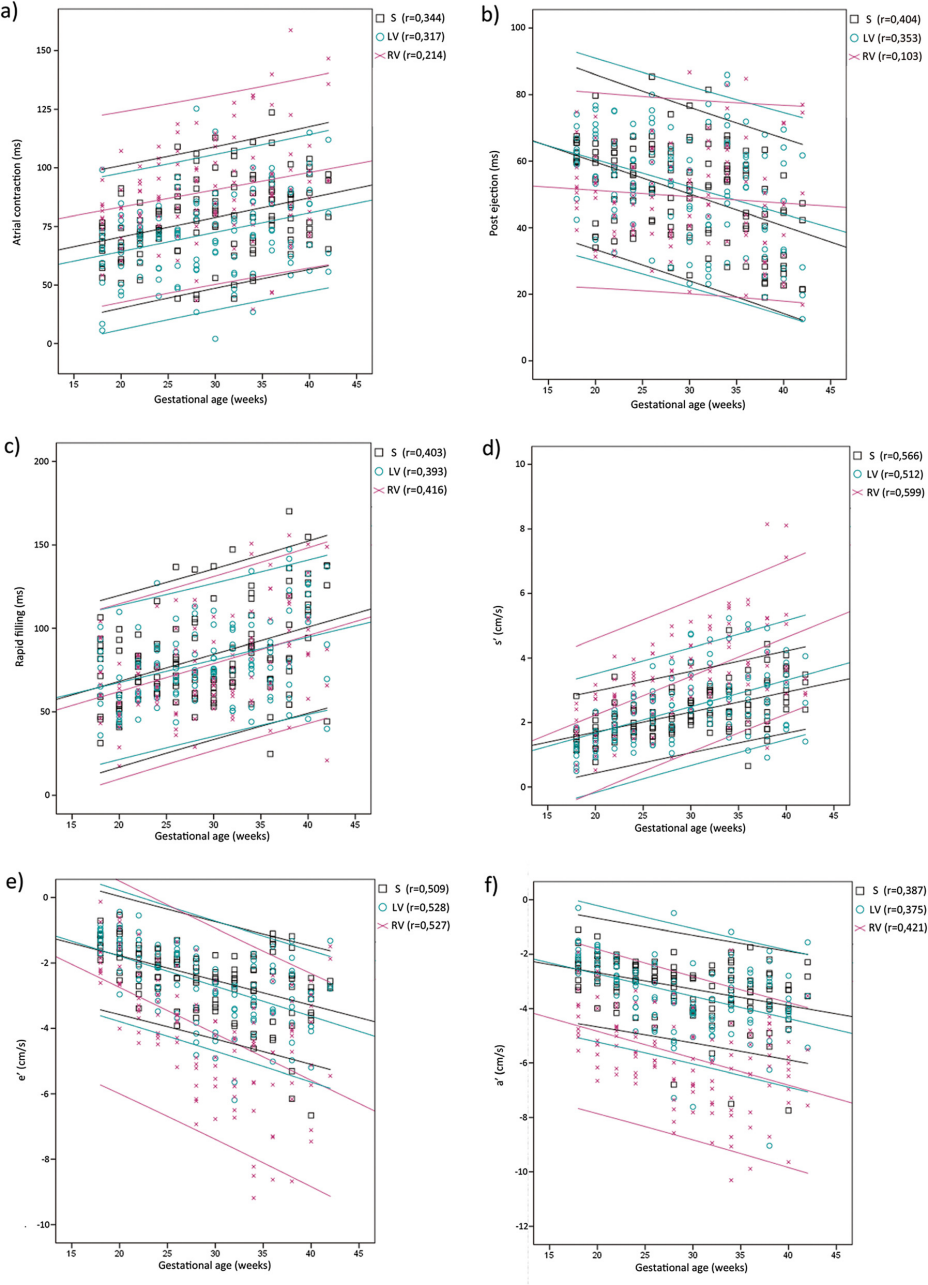
**Tissue velocity imaging reference values.** The duration of **(a)** atrial contraction, **(b)** post ejection and **(c)** rapid filling, as well as the peak velocities of **(d)** ventricular ejection **(s’), (e)** rapid filling **(e’)** and **(f)** atrial contraction **(a’)**, extracted from septum as well as the left and right ventricular free wall plotted over gestational age (p < 0,001). The figure displays linear regression analysis with 95% confidence interval. S, septum; LV, left ventricular free wall; RV, Right ventricular free wall.

Fetal heart rate decreased from a mean 147 bpm (range 133 to 164) at 18 weeks of gestation to a mean 137 bpm (range 117 to 142) at term (r = 0.401), equivalent to a prolonged duration of a fetal cardiac cycle with about 30 ms throughout gestation. This corresponded to an increase in diastolic duration, as the duration of systole did not vary significantly with gestational age, either during pre ejection or ventricular ejection. Figure 3 shows two CSD generated from septum from the average of 10 fetuses at 18 weeks of gestation and 10 fetuses at term, respectively, displaying the different time intervals during a cardiac cycle. During diastole, the duration of atrial contraction and rapid filling were prolonged while the duration of post ejection was shortened. The linear regression plots for the measurements of these phases, made from septum and the left and right ventricular free wall, are displayed over gestational age in Figure 4(a), (b) and (c). Adherent gestational age specific mean values and confidence intervals are stated in Table 3.

Multiple regression analysis showed that a combination of HR and gestational age enhanced the correlations for the duration of artial contraction (r = 0,461) and rapid filling (r = 0,516). However, no significant relationship was observed between HR and the post ejection phase or the systolic durations. Sex was not decisive for any of the measured variables even though HR was observed to be significantly higher for the female fetus up until 30 weeks of gestation.

During a cardiac cycle the longitudinal peak velocities of septum showed a linear increase with gestational age, as did the peak velocities of the left and right ventricular free wall, except for the peak velocity of post ejection (post’). Figure 4(d), (e), (f) show the increase throughout gestation for the peak velocity of ventricular ejection (s’), rapid filling (e’) and atrial contraction (a’) extracted from septum as well as the left and right ventricular free wall. Adherent gestational age specific mean values and confidence intervals are stated in Table 3. The velocity during pre and post ejection show a biphasic motion pattern, but single phase movements may occur as well (more common during pre ejection). However, the negative peak velocities of these phases did not change significantly throughout gestation.

**Figure 5:**
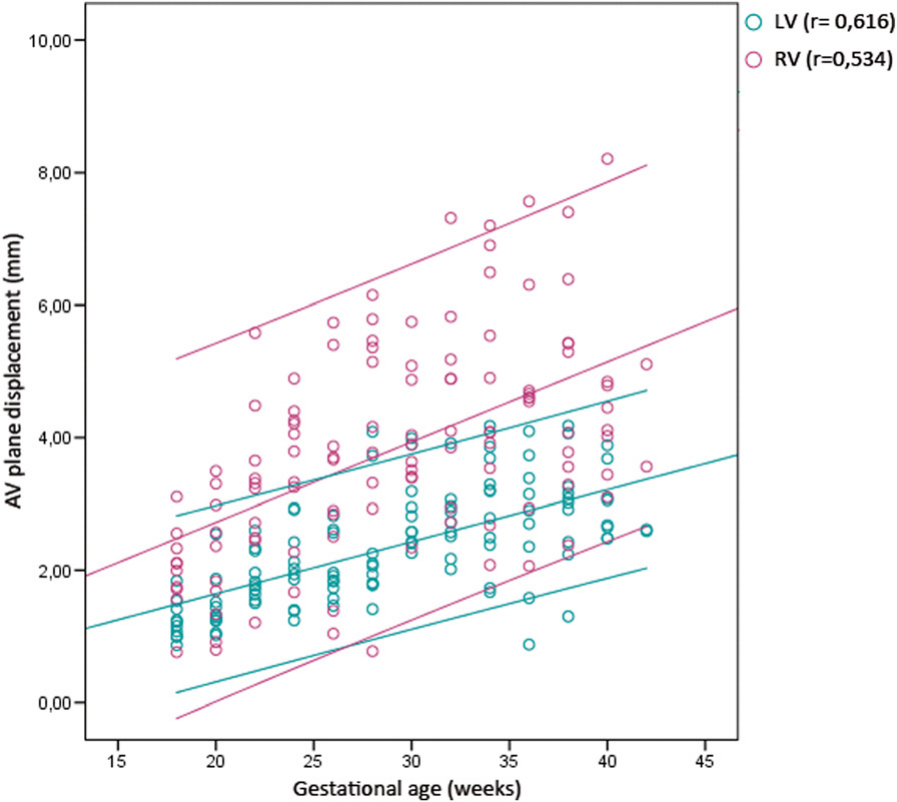
**AV-piston displacement.** The displacement of the atrioventricular plane (AV-piston) increased with gestational age. The figure displays a linear regression analysis with 95% confidence interval for the stroke lengths of the left and right ventricle (p < 0,001). LV, Left ventricle; RV, Right ventricle.

The peak velocities of the right ventricular free wall were higher compared to the peak velocities of septum or the left ventricular free wall, and showed a higher rate of change with gestational age during ventricular ejection and rapid filling. This corresponded to a more prominent increase of the e’/a’ ratio observed in the right ventricular free wall compared to the left ventricular free wall and septum. The displacement of the AV-piston, i.e. the distance covered during ventricular ejection, also showed a linear increase more prominent in the right ventricular free wall as pregnancy advanced, as can be seen in Figure 5. The mean value of the left ventricle (septum and the left ventricular free wall) increased from 1 to 3 mm (r = 0,616), and that of the right ventricular free wall increased from 2 to 5 mm (r = 0,534). The velocity and displacement measurements extracted from the right ventricular free wall consequently displayed a wider distribution compared to septum and the left ventricular free wall.

We could confirm the feasibility and potential use of the new technique, previously demonstrated in a study of non ST-elevation myocardial infarction in adults [11] and a study of experimental hypoxia in fetal sheep [14].

## Discussion

The objective of this study was to establish gestational age specific reference data for TVI measurements of the fetal myocardium. These data also reveal some new information regarding prenatal cardiac function as this study is the first to assess myocardial mechanics during fetal development by evaluating the different time intervals during a cardiac cycle according to the motion shifts of the AV-piston. The finding that the post ejection phase, measured from septum and the left ventricular free wall, decreased with gestational age, might reflect lactate dependent myocardial performance. During this phase the ventricular myocardium starts to relax, which paradoxically is an energy consuming process, caused by the repolarization process of the heart muscle cells, and consequently this phase will be very sensitive to low oxygen levels. In accordance adults exhibit a prolongation of post ejection during exercise and controlled hypoxia resulting in lactecaemia [11]. In a recent study on fetal lamb we also observed a prolongation of post ejection at controlled hypoxia [14], suggesting an adaptive mechanism of the fetal heart to resolve to a more immature state at relative hypoxia. In the right ventricular free wall no significant change of the post ejection phase was observed throughout gestation, which is consistent with the absence of this phase for the corresponding cardiac wall in adults. The prolongation of the rapid filling phase throughout gestation is most likely due to an increase in size and volume. However, the maturational rate of e’ was more prominent, which in accordance with recent findings [15] indicate an improvement in active relaxation as pregnancy advances.

In concurrence with previous studies [2,15-20] we observed that all peak velocities during the cardiac cycle were higher in the right ventricular free wall compared to septum and the left ventricular free wall - consistent with right ventricular dominance. As can be seen in Figure 4, the right ventricle also showed a greater stroke length compared to the left. We could also confirm the gestational increase in fetal myocardial velocities despite a maturational fall in fetal heart rate [7], and the increase of the e’/a’ ratio, corresponding to a redistribution from A-wave towards E-wave dominance with gestational age. The higher ventricular filling velocities confirming the active Frank-Starling mechanism in the fetal heart [7], which is particularly apparent during fetal arrhythmias [21]. The maturational rate of s’ and e’ was higher in the right ventricular free wall compared to the left ventricular free wall and septum, implying that the differential loading of the ventricles does influence the measures of myocardial maturation, unlike Gardiner et al. previously suggested [7]. Sex was not decisive for any of the measured variables even though HR was observed to be significantly higher for the female fetus up until 30 weeks of gestation. Unlike Bilardo et al [22]., we did not observe a relationship between sex and e’, however, multiple regression analysis showed that a combination of HR and gestational age enhanced the correlation for the rapid filling phase. This would imply that an increased HR might affect the ability to measure e’ accurately.

Previous studies that used spectral Doppler reported higher peak velocities compared to the present results [2,19,20,23], which is in agreement with the reported overestimation of velocity using this technique [10]. Also the e’/a’ ratio was higher in these studies, indicating a larger overestimation of e’. Using TVI, different studies have reported varying results [15-18,24]. The measurements of e’ and a’ in the present study were in better compliance with previously reported results than the measurements of s’. An explanation for the observed discrepancies could be the angle dependency of the velocity measurements [25] or the placement of the ROI [25,26]. However, all aforementioned studies state acceptable reproducibility of their measurements. Which is also valid for the measurements made in this study, where reproducibility as well as intra- and inter-observer variability has been demonstrated to be acceptable in the controlled clinical setting [27], and may indicate that the equipment used is not interchangeable - previously demonstrated in both adult [28] and fetal [29] echocardiography. This implies that one should need to establish separate reference materials when measuring amplitudes of the myocardial velocity curve depending on which ultrasound system and is being used. A study demonstrating normal values and age related changes of pre’ also suggest that pre’ and post’ are more reliable than s’, as s’ has shown to be preload and afterload dependent [23,30,31]. Even though we demonstrate less distribution for pre’, the peak velocity during such a short time interval is easy to miss. Further studies are needed in order to confirm the reasons for the discrepancies, and to establish whether this also applies for the timing of the mechanical events during a cardiac cycle.

As long as the timing of myocardial events can be represented accurately, time intervals could be more robust measurements than myocardial velocities as these measurements are less angle dependant and less influenced by tethering effects. Furthermore, time intervals have been described as markers of cardiac performance, where the myocardial performance index or Tei index, reflecting the relationship between the transition phases (pre and post ejection) and ventricular ejection, has been evaluated as a marker of fetal cardiac dysfunction [32-34]. These time intervals were in the present study shown to be independent of heart rate within the studied heart rate interval. However, there was no gestational age related change of this index, despite a maturational shortening of the post ejection phase, which implies that the Tei index might not be as sensitive. Traditionally this index has been calculated from Doppler derived blood flow measurements, which are not comparable to tissue velocity measurements [35], and these measures should not be used interchangeably.

The transition phases mirror the hearts mechanical function and have been shown to be lactate sensitive markers for asphyxia and metabolic acidosis [14,36]. Lactate is a metabolite in anaerobic metabolism and reflects tissue hypoxia. Determination of lactate in blood from the fetus’s scalp during labor has been studied extensively and comparative studies of pH have shown that lactate analysis has similar or better predictive properties compared with pH analysis in the identification of short term neonatal morbidity [37,38]. Thus non-invasive methods to determine the fetal lactate status is of great potential value in the area of fetal monitoring.

The timing of mechanical events has hitherto been difficult to ensure without a simultaneous ECG signal. To make the analysis of TVI measurements easier and more effective we introduced the CSD as a potential aid in fetal monitoring. Using this method we measure the time intervals according to the changes from static to dynamic work based on the DAPP-technology, and thus no longer require a simultaneous ECG signal. This means that in the future we can potentially provide a fully automated evaluation of the longitudinal myocardial velocity profile. Hypothetically this method could give early indications of myocardial dysfunction that should be beneficial in many patient groups where fetal myocardial function is affected and facilitate detection of risk pregnancies as well as aid in the assessment before and after intrauterine fetal therapy.

The provided reference material demonstrated the feasibility of the technique and that the measurements are sensitive enough to yield insight into maturational changes in myocardial function, which provides a foundation that will enable further investigations and potentially allow identification of fetuses with myocardial dysfunction.

## Competing interests

The authors declare that they have no competing interests.

## Authors’ contributions

NE, BL, LÅB and MW participated in the design of the study and the interpretation of data, as well as revised the maniscript. NE performed the offline measurements, statistical analysis and drafted the manuscript. JJ performed the software development, defined the timing of the mechanical events during a cardiac cycle, participated in the interpretation of data and revised the manuscript. KFW and LH performed the ultrasound image acquisition ande revised the manuscript. All authors read and approved the final manuscript.
